# Cross-Talk of Multiple Types of RNA Modification Regulators Uncovers the Tumor Microenvironment and Immune Infiltrates in Soft Tissue Sarcoma

**DOI:** 10.3389/fimmu.2022.921223

**Published:** 2022-07-04

**Authors:** Lin Qi, Wenchao Zhang, Xiaolei Ren, Ruiling Xu, Zhimin Yang, Ruiqi Chen, Chao Tu, Zhihong Li

**Affiliations:** ^1^ Department of Orthopedics, The Second Xiangya Hospital, Central South University, Changsha, China; ^2^ Hunan Key Laboratory of Tumor Models and Individualized Medicine, The Second Xiangya Hospital, Changsha, China; ^3^ Department of Microbiology, Immunology & Molecular Genetics, UT Health Science Center, University of Texas Long School of Medicine, San Antonio, TX, United States

**Keywords:** RNA modification regulator, soft-tissue sarcoma, tumor microenvironment, immune infiltrate, drug sensitivity, immunotherapy

## Abstract

**Background:**

Soft-tissue sarcoma (STS) represents a rare and diverse cohort of solid tumors, and encompasses over 100 various histologic and molecular subtypes. In recent years, RNA modifications including m^6^A, m^5^C, m^1^A, and m^7^G have been demonstrated to regulate immune response and tumorigenesis. Nevertheless, the cross-talk among these RNA modification regulators and related effects upon the tumor microenvironment (TME), immune infiltrates, and immunotherapy in STS remain poorly understood.

**Methods:**

In this study, we comprehensively investigated transcriptional and genetic alterations of 32 RNA modification regulators in STS patients from The Cancer Genome Atlas (TCGA) cohort and validated them in the Gene Expression Omnibus (GEO) cohort. Single-cell transcriptomes were introduced to identify regulators within specific cell types, with own sequencing data and RT-qPCR conducted for biological validation. Distinct regulator clusters and regulator gene subtypes were identified by using unsupervised consensus clustering analysis. We further built the regulator score model based on the prognostic regulator-related differentially expressed genes (DEGs), which could be used to quantitatively assess the risk for individual STS patients. The clinical and biological characteristics of different regulator score groups were further examined.

**Results:**

A total of 455 patients with STS were included in this analysis. The network of 32 RNA modification regulators demonstrated significant correlations within multiple different RNA modification types. Distinct regulator clusters and regulator gene subtypes were characterized by markedly different prognoses and TME landscapes. The low regulator score group in the TCGA-SARC cohort was characterized by poor prognosis. The robustness of the scoring model was further confirmed by the external validation in GSE30929 and GSE17674. The regulator score was negatively correlated with CD4+ T cell, Th2 cell, and Treg cell recruitment and most immunotherapy-predicted pathways, and was also associated with immunotherapy efficacy.

**Conclusions:**

Overall, our study is the first to demonstrate the cross-talk of RNA modification regulators and the potential roles in TME and immune infiltrates in STS. The individualized assessment based on the regulator score model could facilitate and optimize personalized treatment.

## Introduction

Soft-tissue sarcoma (STS) represents a rare and diverse cohort of solid tumors accounting for merely 1% of all adult cancers (1). STS mainly arises from the embryonic mesoderm and encompasses over 100 various histologic and molecular subtypes (2, 3). Previous studies demonstrated that frequently mutated genes including TP53, NF1, and PIK3CA were associated with the prognosis of STS, which suggested potential therapeutic targets (4). In addition, epigenetic regulation also plays a crucial role in tumorigenesis of mesenchymal tumors (5). In recent years, RNA modifications have received increased attention due to their significant effect on gene expression, including DNA transcription to mRNA translation (6, 7).

All RNA bases are capable of hosting different chemical modifications, and RNA modification may contribute to the initiation and development of human diseases (7). Currently, there are over 170 known RNA modifications including but not limited to N^6^-methyladenosine (m^6^A), 5-methylcytosine (m^5^C), N^1^-methyladenosine (m^1^A), 7-methylguanosine (m^7^G), pseudouridine (Ψ), and adenosine-to-inosine RNA editing (A-to-I editing) (8). In most RNA modifications such as m^6^A and m^5^C, the process of modification was mediated by the regulator proteins including writers (methyltransferases), readers (binding proteins), and erasers (demethylase) (7, 9, 10). Due to the modulation of RNA metabolism and protein synthesis, RNA modification regulators mediate tumorigenesis on aspects of cell proliferation, differentiation, and pharmacoresistance (11, 12).

The modification of m^6^A was first found within poly(A) RNA fractions in the 1970s, but growing interests have been paid to this field only from the 2010s with methylated RNA immunoprecipitation-sequencing (MeRIP-Seq) introduced (13, 14). The combination of the next-generation sequencing technology and immunoprecipitation could efficiently map this RNA modification throughout the transcriptome (15). The m^6^A methyltransferase complexes are mainly composed of methyltransferase-like 3 (METTL3), METTL14, METTL16, Wilms’ tumor 1-associated protein (WTAP), zinc finger CCCH-type containing 13 (ZC3H13), RNA-binding motif protein 15 (RBM15), and the corresponding paralogue RBM15B (9). However, m^6^A demethylases including fat mass and obesity-associated protein (FTO) and α-ketoglutarate-dependent dioxygenase alkB homolog 5 (ALKBH5) were initially reported only in the recent decade (16, 17). Reader proteins including YT521-B homology (YTH) family and the insulin-like growth factor 2 mRNA-binding proteins (IGF2BP) family could bind m^6^A-modified mRNA to execute biological functions (18, 19). Significantly, several m^6^A regulators were reported to be abnormally expressed in human tumor tissues, thus initiating tumorigenesis and metastasis (20).

The presence of m^5^C, a methylated form in the fifth carbon of cytosine, could occur in both DNA and RNA (21). However, the function of m^5^C in RNA is less studied. The formation of m^5^C is primarily introduced by the methyltransferase NOP/SUN (NSUN) protein family, including seven members in human (22, 23). Among them, NSUN2 mainly mediates m^5^C formation in mRNA (10, 24). Aly/REF export factor (ALYREF) and Y box binding protein 1 (YBX1) act as the readers binding to the mRNA m^5^C site (10, 24, 25). Increasing evidence indicates that m^5^C is associated with various cellular activities and tumorigenesis (26).

Studies on the modification of m^1^A in mRNA have been gradually expanding since the advent of relevant sequencing technologies in recent years (27, 28). Modification regulators including tRNA methyltransferase 6/61A (TRMT6/61A), TRMT61B, and TRMT61C are currently known writers in m^1^A (29–31). Similar to the modification of m^6^A, YTH family members could mediate the m^1^A process by binding to the corresponding mRNA sites (32). Also, similar to ALKBH5 in m^6^A, methyl groups could also be erased by m^1^A demethylases including ALKBH1 and ALKBH3 (28, 33). The recent study suggested a specific association between m^1^A regulators and cell proliferation in gastrointestinal cancers (34).

Moreover, m^7^G is another significant RNA modification required in most processes of the life cycle of RNA (35). The internal m^7^G methylation is regulated by METTL1 and WD repeat domain 4 (WDR4) (36). Likewise, RNA guanine-7 methyltransferase (RNMT) could also catalyze the methylation at the guanine N7 position, which is associated with tumor growth (7, 37).

However, the above-mentioned studies only focused solely on specific RNA modifications, while the cross-talk among different patterns of RNA modification regulators in STS remain unclear. In the present study, we comprehensively investigated the cross-talk among RNA modification regulators including m^6^A, m^5^C, m^1^A, and m^7^G in the STS of The Cancer Genome Atlas (TCGA) and Gene Expression Omnibus (GEO) cohort. Several RNA modification regulator-related patterns were identified, and relative tumor microenvironment (TME) characteristics were intensively studied. Moreover, we established the prognostic regulator-related scoring model for individual STS patients. The RNA modification regulator-related score could help predict chemoimmunotherapy response in STS patients. The findings demonstrated that the cross-talk among different patterns of RNA modification regulators potentially contributed to shaping TME and immune characteristics, which had significant implications for therapeutic guidance.

## Methods

### Collection and Processing of STS Datasets

**Figure 1:**
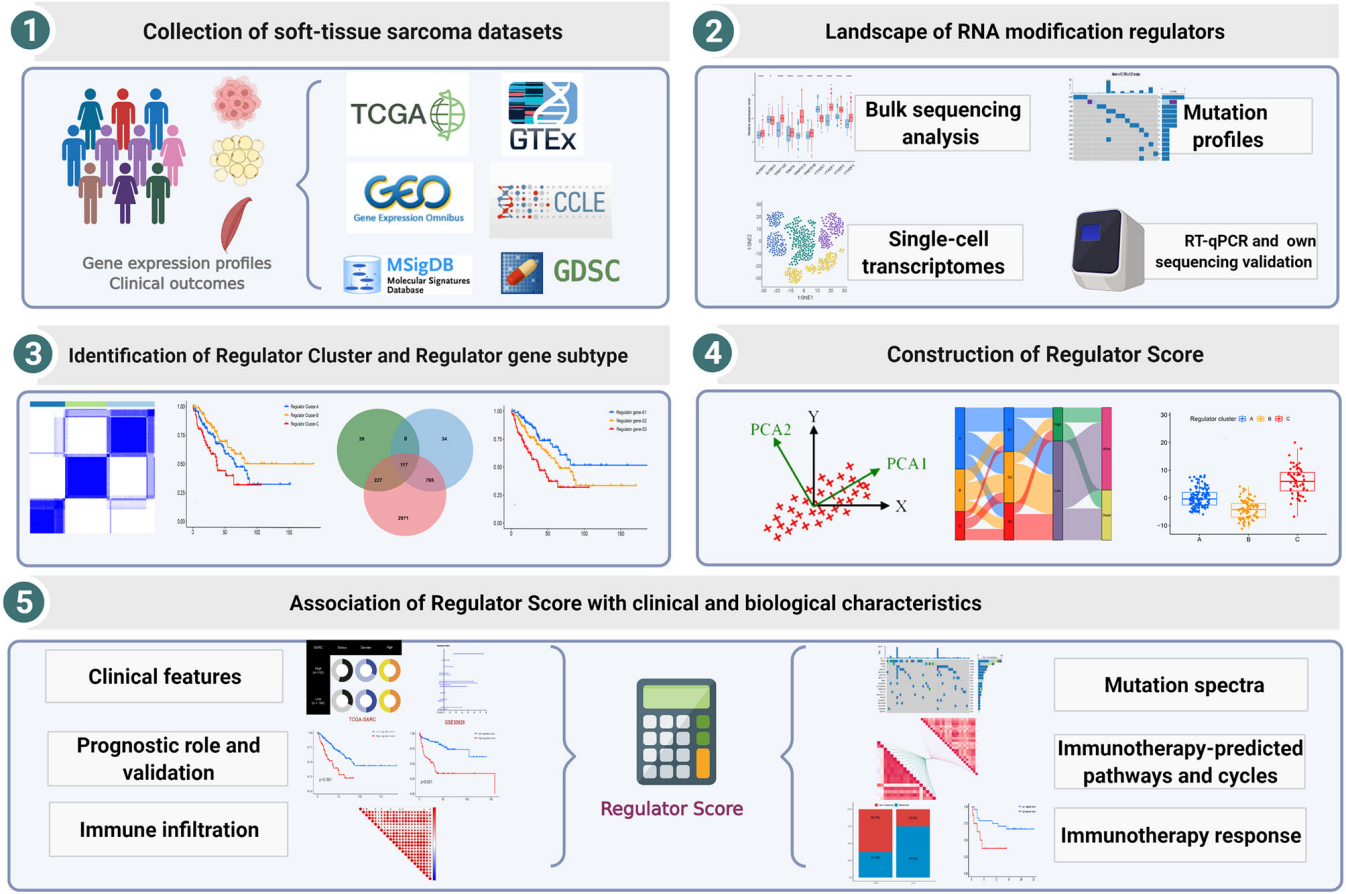
Workflow of this study design.

Overall study workflow is presented in **Figure 1**. We downloaded gene expression profiles of STS and clinical data corresponding to the samples from the TCGA and GEO databases. The expression matrices of normal adipose and muscle tissue were derived from the Genotype-Tissue Expression (GTEx) database. To maximize compatibility and reduce batch effects between the TCGA and GTEx data, the RNA-Seq data from these two databases were processed and unified following sufficiently rigorous procedures, which consist of the uniform realignment, the quantification of gene expression, and the correction of batch effect (38). All TCGA datasets of RNA sequencing, somatic mutations, copy number variations (CNVs), and clinical data were obtained from the UCSC Xena browser (https://xenabrowser.net/) (39). The mutation data were visualized by utilizing the package “maftools” (version 2.8.0). For the GEO database, two eligible STS cohorts with prognosis data (GSE30929 and GSE17674) and one cohort with single-cell RNA-seq data (GSE131309) were collected for further validation analysis. In total, 455 patients with STS were included in our analysis, and basic information is summarized in **Supplementary Table 1**.

For pan-cancer analysis, the TCGA pan-cancer FPKM RNA-seq and clinical data were downloaded *via* the UCSC Xena Browser. The pan-cancer mutation annotation files were obtained from the GDC data portal (https://portal.gdc.cancer.gov/). Furthermore, we also introduced the immunotherapy-treated cohort. The cohort of melanoma patients treated with the combination of anti-PD-1 and anti-CTLA-4 was used to evaluate the association between the regulator score and prognosis after immunotherapy (40).

### Unsupervised Clustering of RNA Modification Regulators

On the basis of prior studies (8, 9, 24, 41–43), a total of 32 RNA modification regulators including m^6^A, m^5^C, m^1^A, and m^7^G were included in the current study (**Supplementary Table 2**). The distribution landscape of selected RNA modification regulators on human chromosomes was plotted by the package “Rcircos” (version 1.2.1). The unsupervised clustering analysis was conducted to recognize RNA modification regulator and gene-related patterns. The R package “ConsensusClusterPlus” (version 1.56.0) was utilized with the key parameters including maxK = 9 and repetitions = 1000, so as to stabilize the identification (44).

### Identifying Differentially Expressed Genes Between Clusters

As distinct RNA modification regulator patterns were identified *via* the unsupervised clustering, we further conducted differential gene expression analysis. The R package “limma” (version 3.48.3) was applied to conduct pairwise comparisons in gene expression among distinct patterns. The lmFit and eBayes functions were utilized to ensure accuracy. Multiple comparisons were corrected by using the Benjamini–Hochberg method (45). The differentially expressed genes (DEGs) were filtered with adjusted *p*-value < 0.05.

### Gene Set Variation Analysis and Gene Ontology Annotation

In order to probe the biological characteristics of different RNA modification regulator-related patterns, gene set variation analysis (GSVA) was conducted by utilizing the R package “GSVA” (version 1.40.1) (46). Similarly, we used GSVA to compare potential biological differences between low and high regulator score subgroups. The priori-defined gene sets (h.all.v7.5.1 and c2.cp.kegg.v7.4) were downloaded from the Molecular Signatures Database (MSigDB). For differential expression analysis of the hallmark gene sets, GSVA output was submitted to the R package “limma” (version 3.48.3) and tested using moderated *t*-statistics. The results were further illustrated as bar chats using the R package “ggplot2” (version 3.3.5). The GO annotation analysis was also conducted by utilizing the R package “clusterProfiler” (version 4.0.5), with false discovery rate (FDR) < 0.05 to determine significant enrichments (47).

The interaction of RNA modification regulator expression in STS was assessed by the Spearman correlation test and visualized by the R package “corrplot” (version 0.90). The network of RNA modification regulator combined with prognostic data was further constructed and visualized by utilizing the R package “igraph” (version 1.2.6).

### Estimation of Cell Infiltration in TME

Single-sample gene set enrichment analysis (ssGSEA) was utilized for quantifying specific immune cell infiltration. Marker genes of specific immune cell types for ssGSEA were retrieved from the published study (48). Levels of immune cell infiltration were normalized ranging from 0 to 1. To investigate the association between TME and potential biological processes, we applied the robust tumor mutation burden (TMB) signatures obtained from published data (49). Moreover, we also calculated ESTIMATE scores of samples, a gene signature-based algorithm that estimates stromal and immune infiltration, by using the R package “ESTIMATE” (version 1.0.13) (50).

Furthermore, signatures related to immunotherapy-predicted pathways and cancer-immunity cycles were extracted from published studies (**Supplementary Tables 4**, **5**) (51, 52). The cancer-immunity cycles established the guiding frameworks for cancer immunotherapy (51). The whole cycles included 7 steps: cancer antigen release and presentation (steps 1 and 2), T-cell priming and activation (step 3), immune cell recruitment (step 4), immune cell infiltration into tumors (step 5), T-cell recognition of cancers (step 6), and killing of cancer cells (step 7). The method of calculating the activity of these steps was reported previously (53). In this study, the signature scores of immunotherapy-predicted pathways and cancer-immunity cycles were calculated by GSVA mentioned above. We then used the R package “ggcor” (version 0.9.4.3) to compare the correlations between the regulator score and GSVA scores of these gene sets.

### Generation of the Regulator-Related Scoring System

The RNA modification regulator-related scoring system was established as follows. First, distinct RNA modification regulator clusters were identified by the unsupervised clustering, and the overlapping DEGs among these clusters were filtered and selected. Then, the univariate Cox regression analysis was used to estimate the prognostic relevance for each DEG. The significantly prognostic genes were extracted, and the principal component analysis (PCA) was further performed based on these prognostic DEGs. Both PC1 and PC2 of prognostic DEGs were selected to serve as the signature scores. This scoring method has significant strength in focusing on the score of the set with the largest block of well-correlated (or anticorrelated) genes, while downweighing contributions from genes unrelated to most set factors, which was applied in previous studies (54, 55). The formula for the scoring system was as follows: regulator- related score = ∑(*PC*1*_i_* + *PC*2*_i_*) where *i* represents the expression of the final determined prognostic DEGs.

### Single-Cell Transcriptome Analysis

Single-cell RNA-seq data were acquired from one published study (GSE131309) (56). Based on the package “Seurat” (version 4.0.5), the data were analyzed following the standard pipeline, which was explained in detail on the official website (https://satijalab.org/seurat/). In the current study, the quality control (QC) metrics were consistent with those in the published study. We conducted gene expression normalization by LogNormalize (scale factor = 10,000). Then, 2,000 highly variable genes (HVGs) were identified with the FindVariableGenes function. Following the results of the ElbowPlot, 25 PCs were selected. Then, cell clustering and t-distributed stochastic neighbor embedding (t-SNE) were further performed based on the above analysis. Moreover, we used the same labels from the data resource to annotate specific cell clusters, and detailed annotation approaches were present in corresponding parts in that study (56). The gene expression of RNA modification regulators was further visualized.

### Chemotherapeutic Sensitivity Prediction

The Genomics of Drug Sensitivity in Cancer (GDSC) was accessed to collect drug response data (https://www.cancerrxgene.org/downloads/anova) (57). The drug response data spanned 518 compounds that target 24 pathways. Furthermore, there were nearly 1,000 human cancer cell lines within this database. To assess chemotherapeutic sensitivity, IC_50_ and drug sensitivity score were used based on the R packages “pRRophetic” (version 0.5) and “oncoPredict” (version 0.2) (58, 59). Moreover, *p*-value was corrected for multiple comparisons where appropriate.

### Cell Lines and Cell Culture

The human synovial sarcoma cell line (SW-982) and liposarcoma cell line (SW-872) were purchased from Procell Life Science & Technology Co., Ltd. The human skin fibroblast cell line (HSF) was purchased from Fenghui Biotechnology Co., Ltd. The primary human synovial sarcoma cells (hSS-005R) were established as previously described (60). The above cell lines were cultured in Dulbecco’s modified Eagle medium (DMEM) supplemented with 10% fetal bovine serum (FBS) at 37°C in 5% CO_2_ atmosphere.

### Real-Time Quantitative PCR

Total RNA of cell lines was extracted using the RNA Express Total RNA Kit (M050, NCM Biotech, China). The cDNA synthesis was performed by utilizing the RevertAid First Strand cDNA Synthesis kit (K1622, Thermo Fisher Scientific, United States). The RT-qPCR was performed as previously described (60). The sequences of the primers for RT-qPCR are presented in **Table S3**.

### Full-Length Transcriptome Analysis

We validated related gene expression level with our own sequencing data including 4 tumor samples and 4 paired normal tissues. Full-length transcriptome analysis was performed by Biomarker Technologies (Biomarker Technologies Ltd, Beijing, China). All operations were in accordance with Oxford Nanopore Technologies (Oxford Nanopore Technologies, Oxford, United Kingdom). The analysis platform (BMKCloud) performs correlation analysis based on reference sequences and nanopore transcriptome sequencing data.

### Statistical Analysis

Statistical analysis was performed by using R software (version 4.1.0). The Spearman correlation test was conducted to calculate the correlations of RNA modification regulators. For pairwise comparisons, data were compared by utilizing Student’s *t*-tests for parametric comparisons and Wilcoxon signed-rank test for nonparametric comparisons. Similarly, one-way ANOVA and Kruskal–Wallis test were applied when over two groups were analyzed. Survival curve comparison was conducted by Log-rank test. Univariate and multivariate Cox regression were utilized to identify significant prognostic factors, with hazard ratio (HR) and 95% confidence interval (CI) calculated. Receiver operating characteristic (ROC) curves were performed to assess the accuracy of the model by utilizing the R package “timeROC” (version 0.4). The function “surv_cutpoint” of the package “survminer” (version 0.4.9) was repeatedly conducted to determine the optimal cutoff values of the regulator scores in the datasets. The patients in the datasets were further dichotomized into low and high regulator score subgroups. We also used chi-square or Fisher exact tests to compare clinical characteristics between two distinct groups. Statistical significance was assigned with two-sided *p*-value < 0.05.

## Results

### Landscape of RNA Modification Regulators in STS

**Figure 2:**
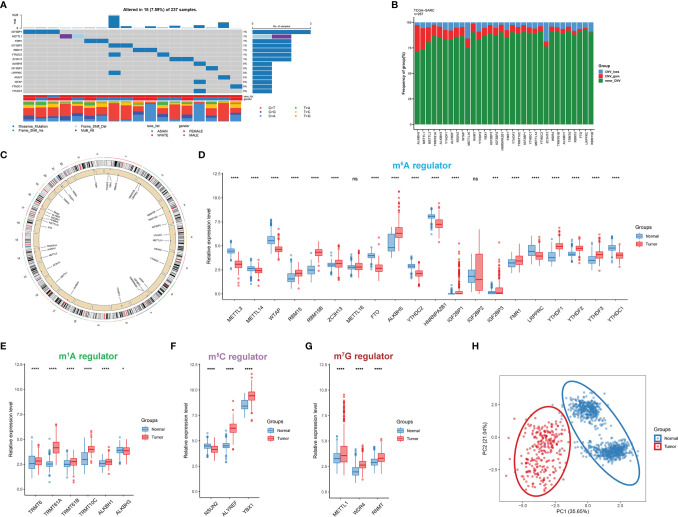
Transcriptional and genetic alterations of RNA modification regulators in STS. **(A)** The mutation frequency of RNA modification regulators in 237 STS patients. The upper bar reflects the TMB. Every single column represents a patient. **(B)** The CNV alteration of RNA modification regulators in STS. The height of the column with a specific color represents the CNV frequency (%). The color represents the CNV status including gain (red) and loss (blue). **(C)** The location of RNA modification regulators on human chromosomes. **(D–G)** The expression level of 32 RNA modification regulators between STS samples (red) and normal tissues (blue) in the TCGA-SARC cohort. The box plot extends from the 25th to 75th percentile and the central line indicates the median. **(H)** Principal component analysis (PCA) of 32 RNA modification regulators for discriminating between tumor and normal samples. ns, p ≥ 0.05; *, 0.01 ≤ p < 0.05; **, 0.001 ≤ p < 0.01; ***, 0.0001 ≤ p < 0.001; ****, p < 0.0001.

In this study, a total of 32 RNA modification regulators were selected. The somatic mutation frequency of RNA modification regulators in STS was first assessed. The mutations were concentrated within 14 RNA modification regulators, and only 18 of 237 STS patients (7.59%) displayed regulator-associated mutations (**Figure 2A**). For a global view in pan-cancer, we also explored the mutation frequency in 32 other cancer types from the TCGA cohort (**Supplementary Figure 1E**). It could be found that the proportion of regulator mutation was relatively low in uveal melanoma (UVM), pheochromocytoma and paraganglioma (PCPG), and testicular germ cell tumors (TGCT), while uterine corpus endometrial carcinoma (UCEC) presented more mutations. The analysis of mutation co-occurrence indicated significant correlations among gene mutations including LRPPRC, IGF2BP2, YTHDC2, ALKBH5, and WTAP (**Supplementary Figure S1A**). However, there was no significant survival difference in overall survival (OS) or disease-free survival (DFS) between STS patients with mutations and without mutations (**Supplementary Figures 1B, C**). Further examination of the CNV alteration indicated that ALKBH5, METTL1, and METTL3 exhibited relatively evident CNV gain, while METTL16 and ZC3H13 presented a relatively substantial proportion of CNV loss (**Figure 2B**). Additionally, the chromosome locations of each RNA modification regulator are depicted in **Figure 2C**. The Gene Ontology (GO) analysis of the regulators indicated that biological processes were mainly enriched in terms of RNA modification (**Supplementary Figure S1D**).

**Figure 3:**
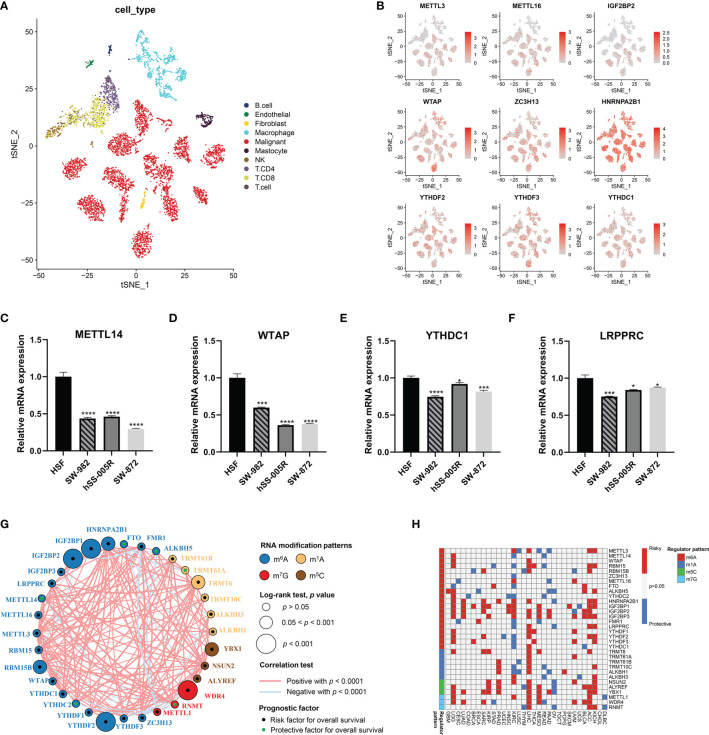
Validation and interaction of RNA modification regulator expression in STS. **(A)** The t-SNE plot demonstrating specific cell clusters. Each color corresponds to one cell type. **(B)** The t-SNE plots illustrating the expression level of specific genes. **(C–F)** Validation of expression of RNA modification regulators in cell lines. **(G)** The interaction of RNA modification regulator expression in STS. The colors represent the types of RNA modification. The size of the circles indicated the prognostic effect assessed by *p*-value. The dots within the circles represent the prognostic roles including protective factor (green) and risk factor (black). **(H)** The prognostic roles of 32 RNA modification regulators across cancers in TCGA. Red indicates the higher regulator expression related to poor survival, while blue suggests the association with good survival. Only statistically significant prognostic factors were present. *, 0.01 ≤ p < 0.05; **, 0.001 ≤ p < 0.01; ***, 0.0001 ≤ p < 0.001; ****, p < 0.0001.

Next, we characterized the gene expression of RNA modification regulators in STS samples against normal tissues. Of 32 RNA modification regulators, 30 regulators were significantly differentially expressed (**Figures 2D–G**). The t-SNE visualization of single cells from STS samples of the GSE131309 dataset was colored by specific cell clusters (**Figure 3A**). The distribution and level of gene expression were also illustrated (**Figure 3B** and **Supplementary Figures 2A, B**). Notably, METTL3, METTL16, and IGF2BP2 mainly clustered in malignant cells, while WTAP, ZC3H13, HNRNPA2B1, YTHDF2, YTHDF3, and YTHDC1 were broadly distributed in all cell clusters. We also conducted RT-qPCR analysis to validate regulator expression in cell lines of STS (**Figures 3C–F**). The expression levels of METTL14, WTAP, YTHDC1, and LRPPRC were significantly lower in STS cell lines including SW-982, hSS-005R, and SW-872, compared with the expression in HSF. The expression level of RNA modification regulators validated by our own sequencing data is shown in **Supplementary Figure 3**. In four pairs of tumor and normal samples, the differences in expression levels of several regulators were consistent with those in public datasets. The prognostic roles of these regulators in TCGA pan-caner datasets were also explored (**Figure 3H**). Furthermore, based on the expression level of 32 RNA modification regulators, we could efficiently discriminate STS samples from normal tissues (**Figure 2H**).

### Identification of RNA Modification Regulator Patterns

The network of 32 RNA modification regulators presented a comprehensive landscape of the interactions (**Figure 3G**). It is noteworthy that the significant correlations were not limited to the single RNA modification but to multiple different RNA modification types. More remarkably, METTL1 showed negative correlation with a substantial proportion of RNA modification regulators. These findings suggested potential cross-talk among these regulators, which might play significant roles in the development of distinct RNA modification patterns.

**Figure 4:**
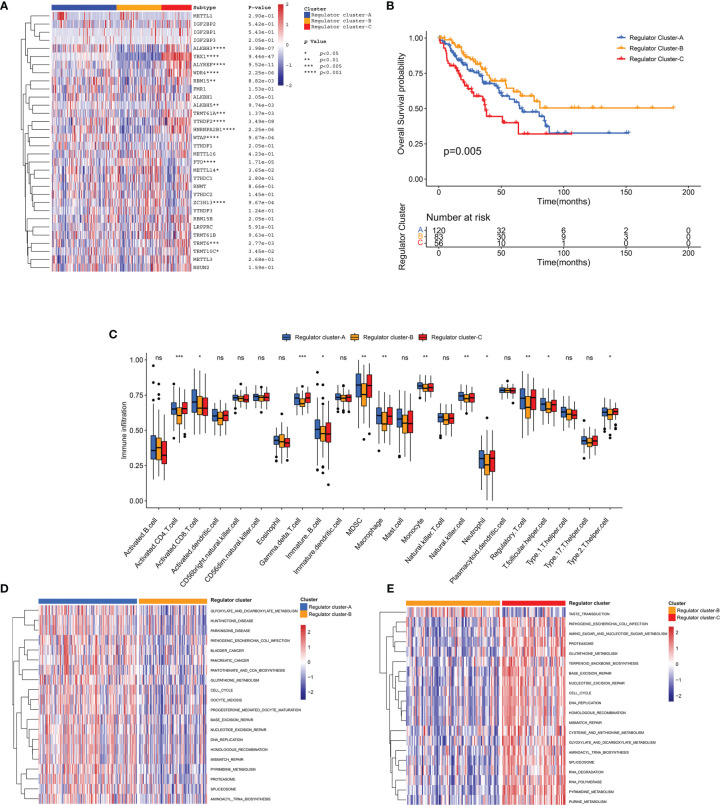
Identification of regulator clusters and related biological characteristics. **(A)** Heatmap of 32 RNA modification regulators among distinct regulator clusters. **(B)** The OS curve comparing survival of the TCGA-SARC cohort in Regulator Clusters A (blue), Regulator Clusters B (orange), and Regulator Clusters C (red). **(C)** The infiltration of immune cells within distinct regulator clusters. The box plot extends from the 25th to 75th percentile, and the central parallel line indicates the median. **(D, E)** The GSVA illustrating pathway enrichment among distinct regulator clusters. ns, p ≥ 0.05; *, 0.01 ≤ p < 0.05; **, 0.001 ≤ p < 0.01; ***, 0.0001 ≤ p < 0.001.

In order to further elucidate distinct RNA modification regulator patterns, unsupervised consensus clustering was conducted to group STS patients in the TCGA-SARC cohort based on the expression of 32 regulators (**Supplementary Figuress 4A–F**). We identified *K* = 3 as the optimal index according to the elbow method (61). Correspondingly, 259 STS patients in the TCGA-SARC cohort were identified into 3 clusters including 120 cases in cluster A, 83 cases in cluster B, and 56 cases in cluster C (**Figure 4A**). These clusters were further named as Regulator Clusters A–C, respectively (**Supplementary Table 6**). Analysis of the survival curve indicated a significant difference among 3 distinct regulator clusters, and Regulator Cluster B showed an apparent survival advantage (**Figure 4B**). For further comparison of pathway enrichment among distinct regulator clusters, GSVA was conducted. As illustrated in **Figures 4D, E** and **Supplementary Figure 4G**, Regulator Cluster C was significantly enriched in pathways associated with DNA replication, mismatch repair, and base excision repair. Of note, further ssGSEA demonstrated that Regulator Cluster C was also enriched with innate immune cell infiltrations that include myeloid-derived suppressor cells (MDSCs), macrophages, monocytes, and natural killer (NK) cells (**Figure 4C**).

### TME Cell Infiltration in Distinct Genomic Subtypes

**Figure 5:**
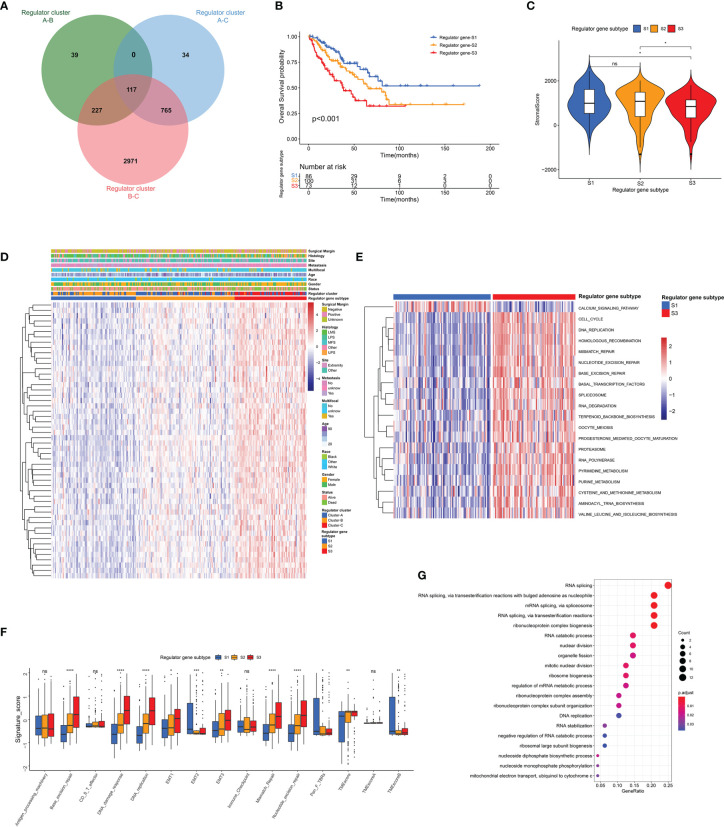
Identification of distinct genomic subtypes and TME cell infiltration. **(A)** Venn diagram showing the overlapping of RNA modification regulator-related DEGs. **(B)** The OS curve comparing survival of the TCGA-SARC cohort in Regulator gene S1 (blue), Regulator gene S2 (orange), and Regulator gene S3 (red). **(C)** Differences in stromal score among three regulator gene subtypes in the TCGA-SARC cohort. **(D)**The unsupervised clustering analysis of the 54 overlapping DEGs in the TCGA-SARC cohort. **(E)** The GSVA illustrating pathway enrichment among distinct regulator gene subtypes. **(F)** Differences in TMB signatures among three regulator gene subtypes in the TCGA-SARC cohort. **(G)** GO enrichment analysis of the 54 overlapping DEGs. ns, p ≥ 0.05; *, 0.01 ≤ p < 0.05; **, 0.001 ≤ p < 0.01; ***, 0.0001 ≤ p < 0.001; ****, p < 0.0001.

To investigate potential associations between TME cell infiltration and RNA modification regulators, the immune cell compositions were compared among distinct patterns (**Supplementary Figure 1F**). As illustrated, most regulators showed negative correlations with CD8^+^ T cells, and were positively correlated with T helper cells and Treg cells. For further exploration of clinical and biological characteristics of distinct regulator clusters, a total of 117 DEGs were identified as RNA modification regulator-related signature, which was illustrated within the overlapping of the Venn diagram (**Figure 5A**). These DEGs were subsequently evaluated by the univariate Cox regression analysis, and 54 DEGs with a prognostic effect were screened out (**Supplementary Table 7**). The main terms in biological processes of GO analysis of these 54 DEGs included RNA splicing, RNA catabolic process, and nuclear division (**Figure 5G**). Consistent with the identification method of regulator clusters, the unsupervised clustering analysis revealed 3 distinct genomic subtypes including Regulator genes S1–S3 based on the 54 DEGs (**Supplementary Figures 5A–F** and **Supplementary Table 6**). We found clear distinction of gene expression among these subtypes, and clinical characteristics were also variable as illustrated (**Figure 5D**). Notably, significant survival differences existed among these regulator gene subtypes, and Regulator gene S3 was correlated with poor prognosis (**Figure 5B**). The stromal scores of Regulator gene S3 were also relatively lower compared with scores in the other two regulator gene subtypes (**Figure 5C**). The pathway enrichment analysis demonstrated significant enrichment for DNA replication, homologous recombination, and mismatch repair in Regulator gene S3 (**Figure 5E** and **Supplementary Figure 5G, H**). Subsequent analysis based on TMB signatures also showed the enhanced activity of base excision repair, DNA damage response, and epithelial–mesenchymal transition (EMT) in Regulator gene S3 (**Figure 5F**). For immune cell infiltration, Regulator gene S3 was enriched with activated CD4^+^ T cells and Th2 cells (**Figure S5I**).

### Construction and Validation of Regulator-Related Score

**Figure 6:**
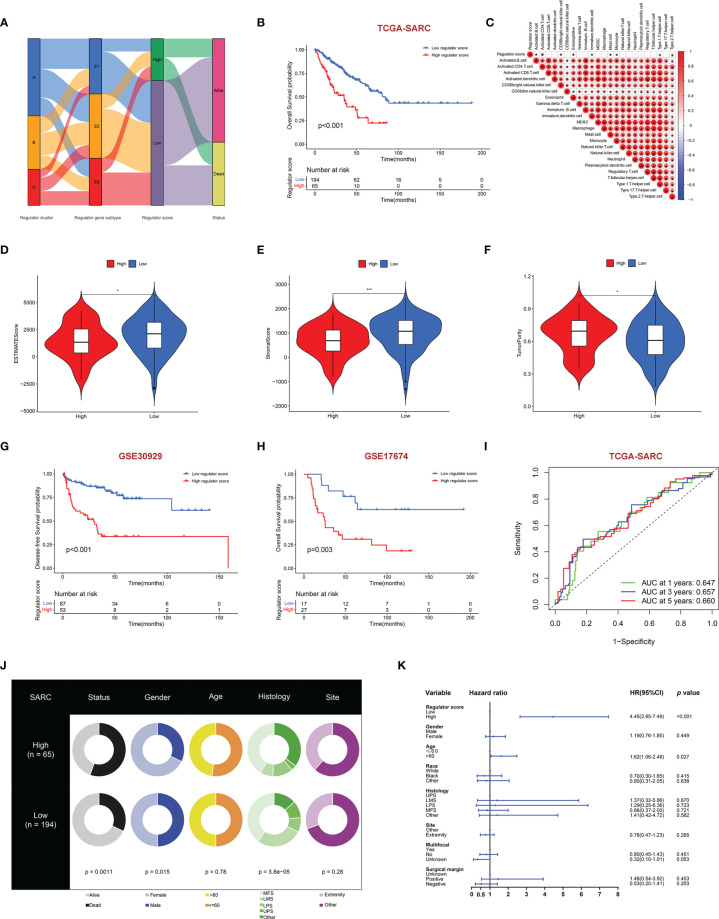
Construction of regulator score and related clinical characteristics. **(A)** Alluvial diagram illustrating the association among regulator clusters, regulator gene subtypes, regulator score groups, and vital status. **(B)** The OS curve comparing survival of the TCGA-SARC cohort in low regulator score (blue) and high regulator score (red) groups. **(C)** Correlations between regulator score and immune cell infiltration by the Spearman correlation test. **(D–F)** Differences of ESTIMATES score **(D)**, stromal score **(E)**, and tumor purity **(F)** between high and low regulator score groups. **(G, H)** The DFS curve of GSE30928 **(G)** and the OS curve of GSE17674 **(H)** in low (blue) and high regulator score (red) groups. **(I)** Time-dependent ROC evaluating the predictive performance of the regulator score in the TCGA-SARC cohort. **(J)** Clinical characteristics between low and high regulator score groups in the TCGA-SARC cohort. **(K)** Multivariate Cox regression of clinical characteristics with regulator score. The horizontal line represents the 95% CI for each variable. The vertical dot line represents the HR of STS patients. *, 0.01 ≤ p < 0.05; **, 0.001 ≤ p < 0.01; ***, 0.0001 ≤ p < 0.001.

Although distinct RNA modification-related regulator clusters and regulator gene subtypes were identified, the analysis was limited within the TCGA-SARC cohort. We further built the RNA regulator score model based on the prognostic regulator-related DEGs, which could be used to calculate for each STS patient. The flow diagram of the development of the regulator score is illustrated in **Figure 6A**. There was a significant difference in the regulator score among distinct regulator gene subtypes (**Supplementary Figure 6A**). The STS patients were further dichotomized into a high regulator score and a low regulator score group according to the cutoff value calculated by the algorithm. It was worth noting that patients with a low regulator score were associated with better prognosis in the TCGA-SARC cohort (*p* < 0.001) (**Figure 6B**). The robustness of the scoring model was further confirmed by the external validation in GSE30929 and GSE17674. The external validation indicated a concordant result in the DFS of GSE30929 (*p* < 0.001) and OS of GSE17674 (*p* = 0.003) with that in the TCGA-SARC cohort (**Figures 6G, H**). The areas under the curve (AUCs) of ROCs for 1-, 3-, and 5-year survival also achieved acceptable values, namely, 0.792, 0.705, and 0.744 in GSE17674, respectively (**Figure 6I** and **Supplementarys Figure 6B, C**). Subgroup analysis of regulator scores in different clinical characteristic groups in the TCGA-SARC cohort also yielded stable results (**Supplementary Figures 6D–N**). In addition, clinical characteristics including gender, age, histology, site, and vital status between the high and low regulator score groups were compared (**Figure 6J**). Multivariate Cox regression analysis was further conducted to determine the prognostic role of the regulator score in STS patients. As illustrated, the regulator score was identified as a robust independent prognostic indicator in the TCGA-SARC cohort (HR = 4.45, 95% CI 2.65–7.49, *p* < 0.001) (**Figure 6K**).

### Association Between Regulator Score and Biological Processes

**Figure 7:**
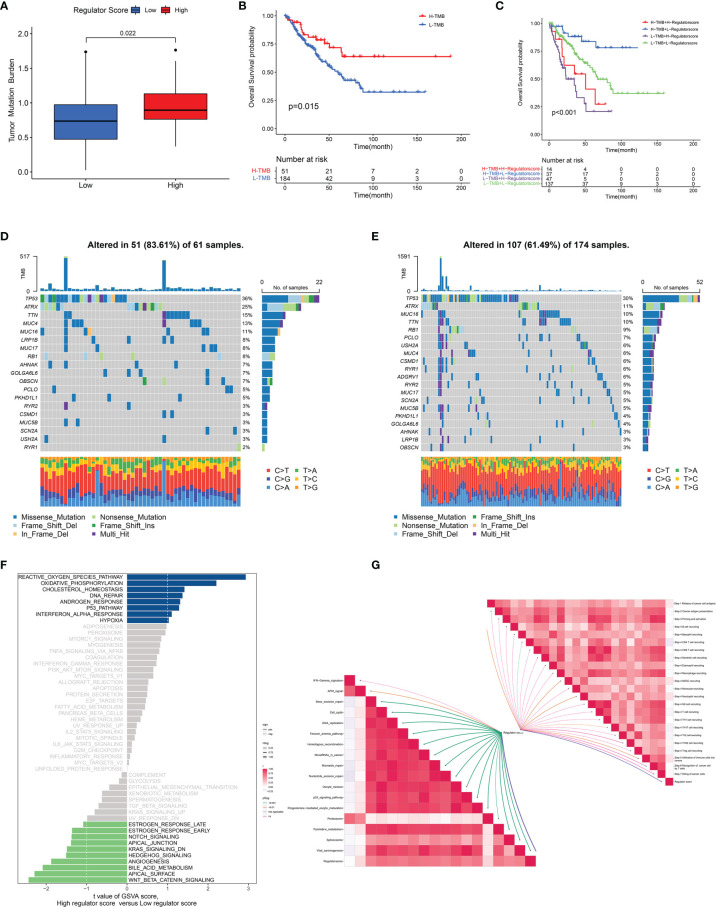
Association between regulator score and biological processes. **(A)** The difference in the TMB level between low and high regulator score groups in the TCGA-SARC cohort. **(B)** The OS curve comparing survival of high- and low-TMB groups in the TCGA-SARC cohort. **(C)** The OS curve illustrating the subgroup analysis of TMB level and regulator score. **(D, E)** The somatic mutation frequency of high **(D)** and low **(E)** regulator score groups in the TCGA-SARC cohort. **(F)** Differences in pathway activities between low and high regulator score groups. **(G)** Correlation of the regulator score with immunotherapy-predicted pathways (lower left) and cancer immunity cycle (upper right).

**Figure 8:**
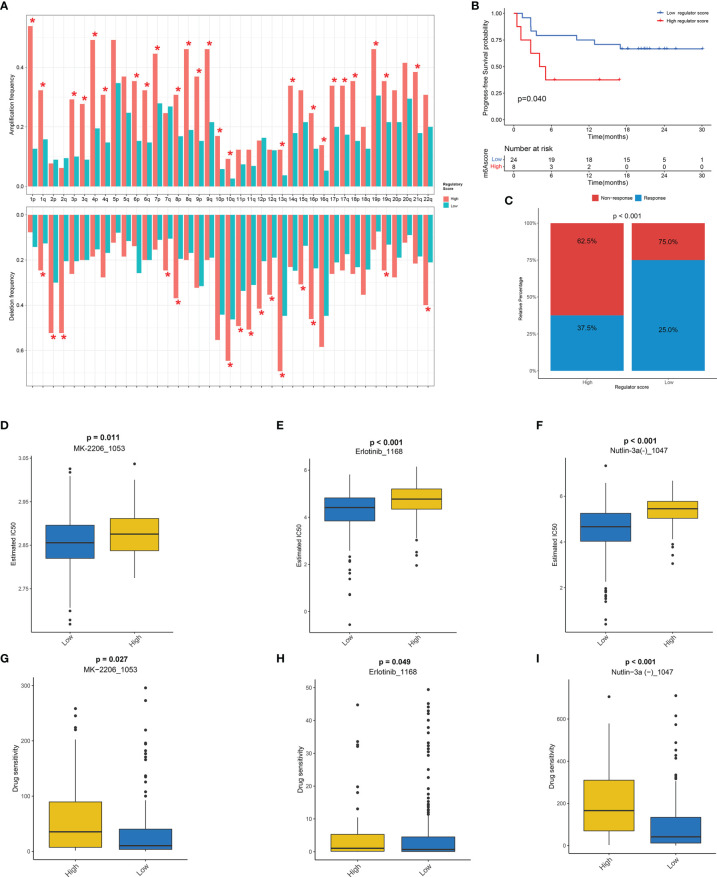
The potential role of regulator score in CNV and chemotherapeutic value. **(A)** The frequency of arm-level amplification and deletion between low and high regulator score groups. **(B)** The progression-free survival (PFS) curve comparing survival of high and low regulator score groups in a cohort of melanoma patients treated with the combination of anti-PD-1 and anti-CTLA-4. **(C)** The proportion of clinical response to anti-PD-1 with anti-CTLA-4 immunotherapy in high and low regulator score groups in the melanoma cohort. **(D–F)** The box plot of the estimated IC50 of MK-2206 **(D)**, erlotinib **(E)**, and Nutlin-3a **(F)** between low and high regulator score groups. **(G–I)** The box plot of the predicted drug sensitivity scores of MK-2206 **(G)**, erlotinib **(H)**, and Nutlin-3a **(I)** between low and high regulator score groups. A lower drug sensitivity score indicated that this group could be more sensitive to the drug therapy. *, p < 0.05.

Because of the significant correlation between the regulator score and the prognosis of STS patients, we further investigated potential biological processes associated with the regulator score. As illustrated in **Figure 6C**, the regulator score exhibited significant inverse correlations with innate immune cells including CD56^bright^ NK cells, eosinophils, immature dendritic cells, mast cells, and monocytes. Moreover, the ESTIMATE score and the stromal score were significantly higher in the STS patients with a low regulator score, while those with a high regulator score obtained a significantly higher tumor purity score (**Figures 6D–F**). Higher TMB was also found in the high regulator score group (**Figure 7A** and **Supplementary Figure 5J**). In the TCGA-SARC cohort, survival analysis demonstrated that the poor prognosis was associated with lower TMB, which would further deteriorate combined with a higher regulator score (**Figures 7B, C**). For the frequency of the somatic mutation between these two groups, we observed more mutations in the high regulator score group with a sample mutation proportion of 83.61%, compared with 61.49% in the low regulator score group (**Figures 7D, E**). It is also noteworthy that the high regulator score group had a significantly higher frequency of arm-level amplification and deletion than the low regulator score group (*p* < 0.05) (**Figure 8A**). When comparing the pathway activities between distinct regulator score groups, we found a considerable increase of reactive oxygen species production and oxidative phosphorylation, but the activity level of the Wnt/β-catenin signaling pathway strongly decreased (**Figure 7F**). As the cancer-immunity cycles were of guiding significance for immunotherapy, the correlation with the regulator score was also explored. There was an inverse correlation among CD4^+^ T cell, dendritic cell, Th17 cell, Th2 cell, and Treg cell recruitment with the regulator score (**Figure 7G**). Meanwhile, the regulator score was negatively correlated with most of the immunotherapy-predicted pathways, indicating its potential role in related immunotherapy.

### Potential Role of Regulator Score in Chemotherapeutic Value

The strong link between the regulator score and TME prompted us to further explore the predictive effect of the regulator score on response to checkpoint immunotherapy. As there was no information about immunotherapy in the TCGA-SARC cohort, a cohort of melanoma patients treated with the combination of anti-PD-1 and anti-CTLA-4 was utilized. The regulator score of an individual patient was calculated on the basis of the scoring system mentioned above. Surprisingly, patients with a low regulator score exhibited significant survival advantage compared with those in the high regulator score group (*p* = 0.040) (**Figure 8B**). Moreover, the response rates to immunotherapy were also significantly higher in the low regulator score group compared with those in high regulator score group (*p* < 0.001) (**Figure 8C**). In addition to immunotherapy, other chemotherapeutic agents might also exert a potential anti-cancer effect. Therefore, the GDSC database was selected because of the large chemotherapeutic agents available. After systematic drug screening, three common chemotherapeutic agents including MK-2206, erlotinib, and Nutlin-3a were identified with IC_50_ varying significantly between the high and low regulator score groups (**Figures 8D–F**). Significant differences in the drug sensitivity score of these drugs were also illustrated between two scoring groups, suggesting that the low regulator score group was more sensitive to these drugs (**Figures 8G–I**).

## Discussion

Numerous studies have demonstrated that RNA modification played an important role in multiple biological processes mediated by various regulators (9, 10, 42, 43). However, considerable studies focused solely on the single type of RNA modification. Furthermore, the overall landscape of TME and immune infiltrates mediated by different patterns of RNA modification regulators have not been studied in depth. Consequently, exploring the cross-talk among RNA modification regulators including m^6^A, m^5^C, m^1^A, and m^7^G in the STS may help elucidate the characteristics of TME and corresponding subtypes and further develop a therapeutic strategy for STS treatment.

Hence, a total of 32 RNA modification regulators were thoroughly studied on the basis of gene expression, mutation patterns, and CNV profiles. The proportion of mutations within regulators were relatively lower, which was consistent with related studies in other malignancies (62, 63). We also demonstrated that the expression levels of RNA modification regulators were significantly different between STS and normal samples. The expression of several RNA modification regulators was verified by utilizing RT-qPCR in cell lines, which may shed creative lights on further research on STS. The emergence of single-cell transcriptomes contributed to identifying gene expression at high resolution within specific cell types (64). Of note, METTL3, METTL16, and IGF2BP2 were mainly represented in the malignant clusters. According to the study focusing on the role of METTL3 in lung adenocarcinoma, METTL3 could enhance mRNA translation including EGFR and Hippo pathways, further promoting growth and invasion of human lung cancer cells (65). In line with this study, the expression level of METTL3 was significantly upregulated in osteosarcoma tissues and cell lines (66). Moreover, silencing METTL3 could suppress tumor proliferation and migration and was also associated with lymphoid enhancer-binding factor 1 (LEF1) and Wnt/β-catenin signaling pathway. Substantial cross-talk among RNA modification regulators was observed, which was consistent with the results in colorectal cancer (67). As a limited number of studies have been conducted concerning the cross-talk of RNA modification regulators, further studies with biological mechanism research are warranted in the future.

Concerning the distinct characteristics of these regulators, three regulator clusters were identified by utilizing the unsupervised consensus clustering. This method could help discover conformational details that might be masked due to population averages, thus identifying potential meaningful patterns (68, 69). Regulator Cluster C was characterized by poor prognosis compared with the other two clusters. It has been demonstrated that MDSCs were relatively enriched in Regulator Cluster C, which has been extensively studied and considered as immunosuppressive cells (70). The proangiogenic capacity of immature myeloid cells may facilitate tumor growth and metastasis (71). Preliminary evidence suggested promising activity when drugs targeting reprogramming of the metabolism of MDSCs were applied in combination with immune checkpoint inhibitors (72). The DEGs on the basis of distinct regulator clusters were mainly enriched in the biological process of RNA splicing, indicating the significant role of RNA modification in regulating RNA splicing, stabilization, and metabolism (20, 73–75). Similar to the analysis of regulator clusters, three regulator gene subtypes were identified with markedly different prognoses and TME landscapes. The stromal scores were significantly reduced in Regulator gene S3, also suggesting the low level of infiltrating stromal cells in this subtype in STS (50).

Although STS cohorts could be identified into distinct clusters based on the robust clustering algorithm, an accurate approach was needed to quantitatively assess RNA modification-related risk for STS patients at the single individual level. The RNA regulator score model established in the current study has significant clinical values and could guide treatment for STS patients. Firstly, the RNA regulator score could serve as a strong prognostic indicator for STS. As we expected, Regulator gene S3 with a relatively poor prognosis also scored significantly higher. Moreover, the RNA regulator score could efficiently distinguish TME characteristics concerning tumor purity, and stromal and immune cell infiltration in individual STS patients. This study found a mild positive correlation between regulator score and TMB. Notably, the relationship between TMB and survival was controversial and varied across tumor types (76, 77). The TCGA-SARC patients with high TMB presented a better prognosis, which needs to be further explored in future studies. Furthermore, a combined prediction model including TMB and the regulator score could provide better outcome prediction. In this study, the potential role of the regulator score in cancer-immunity cycles cannot be ignored, which also suggested that RNA modification could regulate immunotherapy (78). As is widely known, tumor progression was associated with the driver mutations (79). Moderate differences were identified in multiple mutant genes between groups with different regulator scores. We observed a relatively increased mutation rate of ATRX in the high regulator score group, which was characterized by poor prognosis. Previous studies demonstrated that protein coded by ATRX has been implicated in chromatin remodeling at telomeres. Therefore, ATRX mutations may consequently lead to an abnormal telomeric phenotype, which has been proven in glioma (80, 81).

In the absence of an STS cohort receiving immunotherapy, we introduced an independent melanoma dataset treated with the combination of anti-PD-1 and anti-CTLA-4. The strength of the regulator score was further verified while additional prospective studies of STS concerning immunotherapy are still needed for further verification. Additionally, the regulator score-based drug screening could identify potential chemotherapeutic agents for personalized therapy.

There are growing lines of evidence of widespread cross-talk between RNA modification regulators in a wide variety of tumors (82). In colorectal cancer, the interactions of 26 RNA modification regulators could redefine the characteristics of TME and give a more reliable indication of the prognosis (67). Moreover, the importance of noncoding RNAs with RNA modification has become increasingly appreciated in recent years (83, 84). With further study upon RNA modification, more specific regulators have been identified and a total of 32 RNA modification regulators have been included in the current study. Future research should also focus on the cross-talk between RNA modification regulators in non-neoplastic diseases.

In conclusion, this study, for the first time, represented a comprehensive and systematic analysis of four types of RNA modification regulators in STS. The cross-talk of RNA modification regulators played a significant role in regulating the complexity of TME, which was strongly associated with the prognosis of STS patients. The individualized assessment based on the regulator score model could facilitate and optimize personalized treatment. In a broad perspective, this work reinforces the significance of the cross-talk of RNA modification regulators and sheds new light on the individualized treatment strategies for STS patients.

## Data Availability Statement

The datasets presented in this study can be found in online repositories. The names of the repository/repositories and accession number(s) can be found below: NCBI GEO, accession no: GSE198568. https://www.ncbi.nlm.nih.gov/geo/query/acc.cgi?acc=GSE198568.

## Author Contributions

LQ and ZL conceived and designed this study. WZ, XR, LQ, and ZY performed the data analysis, plotting of figures, and writing. RX, RC, and CT were responsible for the critical reading of the manuscript. All authors participated in interpreting the results and revision of the manuscript and approved the submitted version. All authors read and approved the final manuscript.

## Funding

This work was supported by grants from the National Natural Science Foundation of China (NSFC; No. 81902745, No. 82172500, and No. 82103228), the Hunan Provincial Research and Development Program in Key Areas (2020DK 2003), and the China Postdoctoral Science Foundation (No. 2021M693557).

## Conflict of Interest

The authors declare that the research was conducted in the absence of any commercial or financial relationships that could be construed as a potential conflict of interest.

The reviewer YZ declared a shared parent affiliation with the authors LQ, WZ, XR, RX, RC, CT and ZL to the handling editor at the time of review.

## Publisher’s Note

All claims expressed in this article are solely those of the authors and do not necessarily represent those of their affiliated organizations, or those of the publisher, the editors and the reviewers. Any product that may be evaluated in this article, or claim that may be made by its manufacturer, is not guaranteed or endorsed by the publisher.

## Supplementary Material

Supplementary Table 1 Basic information of public datasets included in this study.

Supplementary Table 2 Summary of different RNA modification regulators.

Supplementary Table 3 Sequences of the primers used in RT-qPCR.

Supplementary Table 4 Immunotherapy-predicted pathways signatures.

Supplementary Table 5 Cancer immunity cycles signatures.

Supplementary Table 6 Samples clustering in TCGA-SARC cohort.

Supplementary Table 7 Prognostic analysis of 54 RNA modification regulator-related DEGs using a univariate Cox analysis.

Supplementary Figure 1 Correlation and prognostic analysis of RNA modifications regulators in STS. **(A)** The mutation co-occurrence and exclusion analysis for 14 mutated regulators. The color represents the correlations including co-occurrence (green) and mutually exclusion (purple). **(B)** Overall survival (OS) curve of STS patients with (red) and without mutations (green) of RNA modification regulators. **(C)** Disease-free survival (DFS) curve of STS patients with (red) and without mutations (green)of RNA modification regulators. **(D)** GO enrichment analysis of 32 RNA modification regulators. The horizontal axis represents the gene ratio. **(E)** The mutation frequency of 32 RNA modification regulators in other 32 cancer types of TCGA cohort. The sample size of each cancer type was given in the in bracket. **(F)** The spearman correlation between TME cell infiltration and RNA modification regulators. **(G)** Association of expression of RNA modification regulators with OS based on univariate Cox regression analysis.

Supplementary Figure 2 Expression of RNA modification regulators in STS at single-cell resolution. **(A)** The t-SNE plots illustrating the expression level of specific genes. **(B)** The stacked violin plots demonstrating the expression level of specific genes across cell clusters.

Supplementary Figure 3 The expression level of RNA modification regulators verified by sequencing data. **(A–L)** The expression level of RBM15B, ZC3H13, METTL16, IGF2BP2, FMR1, YTHDF2, TRMT61A, TRMT61B, ALKBH1, ALLYREF, WDR4 and RNMT based on the sequencing data.

Supplementary Figure 4 Analysis of RNA modification Regulator Clusters in STS. **(A–D)** Consensus clustering based on RNA modification regulators (*K* = 2-5). **(E)** Consensus cumulative distribution function (CDF) Plot based on RNA modification regulators. **(F)** Delta area plot of consensus clustering based on RNA modification regulators. **(G)** The GSVA illustrating pathway enrichment among distinct Regulator Clusters.

Supplementary Figure 5 Analysis of Regulator gene subtypes in STS. **(A–D)** Consensus clustering based on regulator-related DEGs (*K* = 2-5). **(E)** The CDF Plot based on the 54 RNA modification regulator-related DEGs. **(F)** Delta area plot of consensus clustering based on the DEGs. **(G, H)** The GSVA illustrating pathway enrichment among distinct Regulator gene subtypes. **(I)** The infiltration of immune cells within distinct Regulator gene subtypes. **(J)** Scatter plots depicting the positive correlation between TMB and Regulator Score in TCGA-SARC cohort.

Supplementary Figure 6 Validation and subgroup analysis of Regulator Score. **(A)** Differences of Regulator Score among distinct Regulator Clusters. **(B, C)** Time-dependent ROC evaluating the predictive performance of Regulator Score in GSE17674 **(B)** and GSE30929 **(C)**. **(D–N)** Subgroup analysis of Regulator Scores in different clinical characteristics groups including age, gender, site and histology.

## Abbreviations

STS, soft-tissue sarcoma; m6A, N6-methyladenosine; m5C, 5-methylcytosine; m1A, N1-methyladenosine; m7G, 7-methylguanosine; Ψ, pseudouridine; A-to-I editing, adenosine-to-inosine RNA editing; MeRIP-Seq, methylated RNA immunoprecipitation-sequencing; METTL3, Methyltransferase Like 3; METTL14, Methyltransferase Like 14; METTL16, Methyltransferase Like 16; WTAP, Wilms’ tumor 1-associated protein; ZC3H13, zinc finger CCCH-type containing 13; RBM15, RNA-binding motif protein 15; FTO, obesity-associated protein; ALKBH5, α-ketoglutarate-dependent dioxygenase alkB homolog 5; YTH, YT521-B homology; IGF2BP, insulin-like growth factor 2 mRNA-binding proteins; NSUN, methyltransferases NOP/SUN; ALYREF, Aly/REF export factor; YBX1, Y box binding protein 1; TRMT6/61A, tRNA methyltransferase 6/61A; TRMT61B, tRNA methyltransferase 61B; TRMT61C, tRNA methyltransferase 61C; WDR4, WD repeat domain 4; RNMT, RNA guanine-7 methyltransferase; TCGA, The Cancer Genome Atlas; GEO, Gene Expression Omnibus; TME, tumor microenvironment; GTEx, the Genotype-Tissue Expression; CNVs, copy number variations; DEGs, differentially expressed genes; GSVA, gene set variation analysis; GO, Gene Ontology; MSigDB, Molecular Signatures Database; FDR, false discovery rate; ssGSEA, single-sample gene set enrichment analysis; TMB, tumor mutation burden; PCA, principal component analysis; GGI, gene expression grade index; QC, quality control; HVG, highly variable genes; t-SNE, t-distributed stochastic neighbor embedding; GDSC, The Genomics of Drug Sensitivity in Cancer; HR, hazard ratio; CI, confidence interval; ROC, receiver operating characteristic; UVM, uveal melanoma; PCPG, pheochromocytoma and paraganglioma; TGCT, testicular germ cell tumors; UCEC, uterine corpus endometrial carcinoma; OS, overall survival; DFS, disease-free survival; MDSC, myeloid-derived suppressor cell; EMT, epithelial–mesenchymal transition; LEF1, lymphoid enhancer-binding factor 1.

## Glossary

**Table:**

STS	soft-tissue sarcoma
m6A	N6-methyladenosine
m5C	5-methylcytosine
m1A	N1-methyladenosine
m7G	7-methylguanosine
Ψ	pseudouridine
A-to-I editing	adenosine-to-inosine RNA editing
MeRIP-Seq	methylated RNA immunoprecipitation-sequencing
METTL3	Methyltransferase Like 3
METTL14	Methyltransferase Like 14
METTL16	Methyltransferase Like 16
WTAP	Wilms' tumor 1-associated protein
ZC3H13	zinc finger CCCH-type containing 13
RBM15	RNA-binding motif protein 15
FTO	obesity-associated protein
ALKBH5	α-ketoglutarate-dependent dioxygenase alkB homolog 5
YTH	YT521-B homology
IGF2BP	insulin-like growth factor 2 mRNA-binding proteins
NSUN	methyltransferases NOP/SUN
ALYREF	Aly/REF export factor
YBX1	Y box binding protein 1
TRMT6/61A	tRNA methyltransferase 6/61A
TRMT61B	tRNA methyltransferase 61B
TRMT61C	tRNA methyltransferase 61C
WDR4	WD repeat domain 4
RNMT	RNA guanine-7 methyltransferase
TCGA	The Cancer Genome Atlas
GEO	Gene Expression Omnibus
TME	tumor microenvironment
GTEx	the Genotype-Tissue Expression
CNVs	copy number variations
DEGs	differentially expressed genes
GSVA	gene set variation analysis
GO	Gene Ontology
MSigDB	Molecular Signatures Database
FDR	false discovery rate
ssGSEA	single-sample gene set enrichment analysis
TMB	tumor mutation burden
PCA	principal component analysis
GGI	gene expression grade index
QC	quality control
HVG	highly variable genes
t-SNE	t-distributed stochastic neighbor embedding
GDSC	The Genomics of Drug Sensitivity in Cancer
HR	hazard ratio
CI	confidence interval
ROC	receiver operating characteristic
UVM	uveal melanoma
PCPG	pheochromocytoma and paraganglioma
TGCT	testicular germ cell tumors
UCEC	uterine corpus endometrial carcinoma
OS	overall survival
DFS	disease-free survival
MDSC	myeloid-derived suppressor cell
EMT	epithelial–mesenchymal transition
LEF1	lymphoid enhancer-binding factor 1.
