# Supplementary figures and images for: Cross-Talk of Multiple Types of RNA Modification Regulators Uncovers the Tumor Microenvironment and Immune Infiltrates in Soft Tissue Sarcoma
Source: Front Immunol. 2022 Jul 4;13:921223. doi: 10.3389/fimmu.2022.921223 (PMC9289169; doi:10.3389/fimmu.2022.921223)

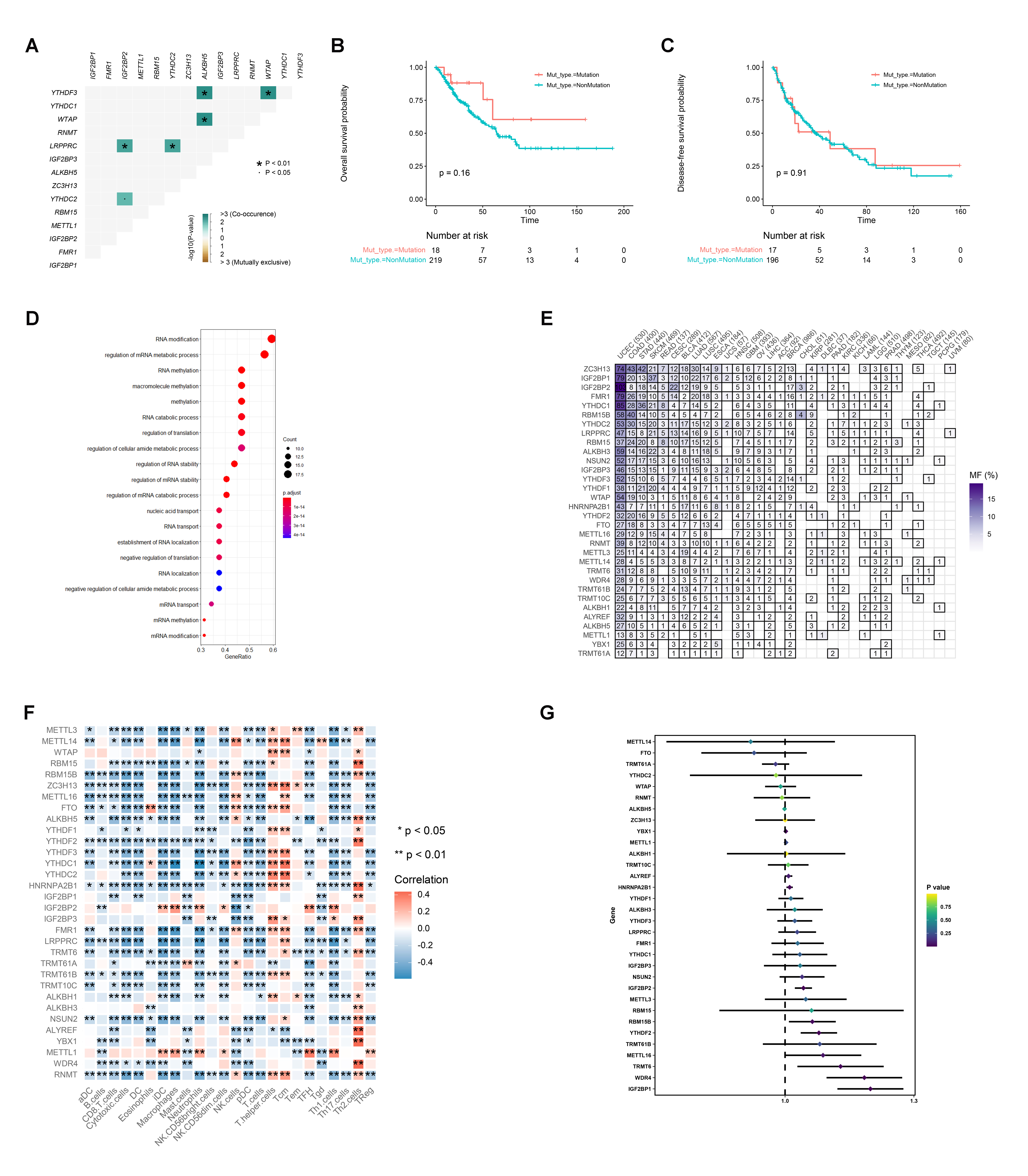

Supplement: Supplementary Figure 1 — Correlation and prognostic analysis of RNA modifications regulators in STS. (A) The mutation co-occurrence and exclusion analysis for 14 mutated regulators. The color represents the correlations including co-occurrence (green) and mutually exclusion (purple). (B) Overall survival (OS) curve of STS patients with (red) and without mutations (green) of RNA modification regulators. (C) Disease-free survival (DFS) curve of STS patients with (red) and without mutations (green)of RNA modification regulators. (D) GO enrichment analysis of 32 RNA modification regulators. The horizontal axis represents the gene ratio. (E) The mutation frequency of 32 RNA modification regulators in other 32 cancer types of TCGA cohort. The sample size of each cancer type was given in the in bracket. (F) The spearman correlation between TME cell infiltration and RNA modification regulators. (G) Association of expression of RNA modification regulators with OS based on univariate Cox regression analysis. [file Image_1.jpeg]

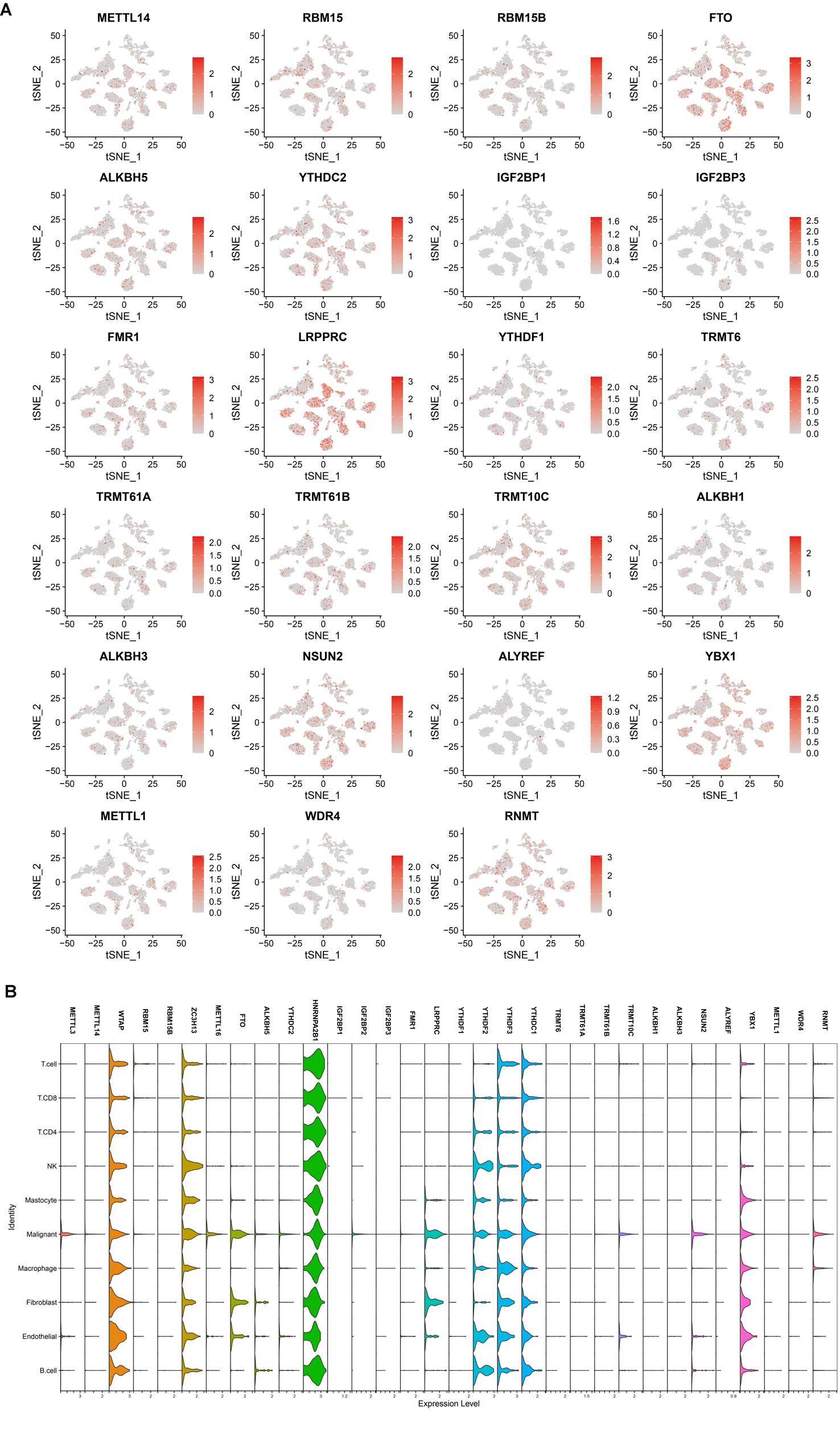

Supplement: Supplementary Figure 2 — Expression of RNA modification regulators in STS at single-cell resolution. (A) The t-SNE plots illustrating the expression level of specific genes. (B) The stacked violin plots demonstrating the expression level of specific genes across cell clusters. [file Image_2.jpeg]

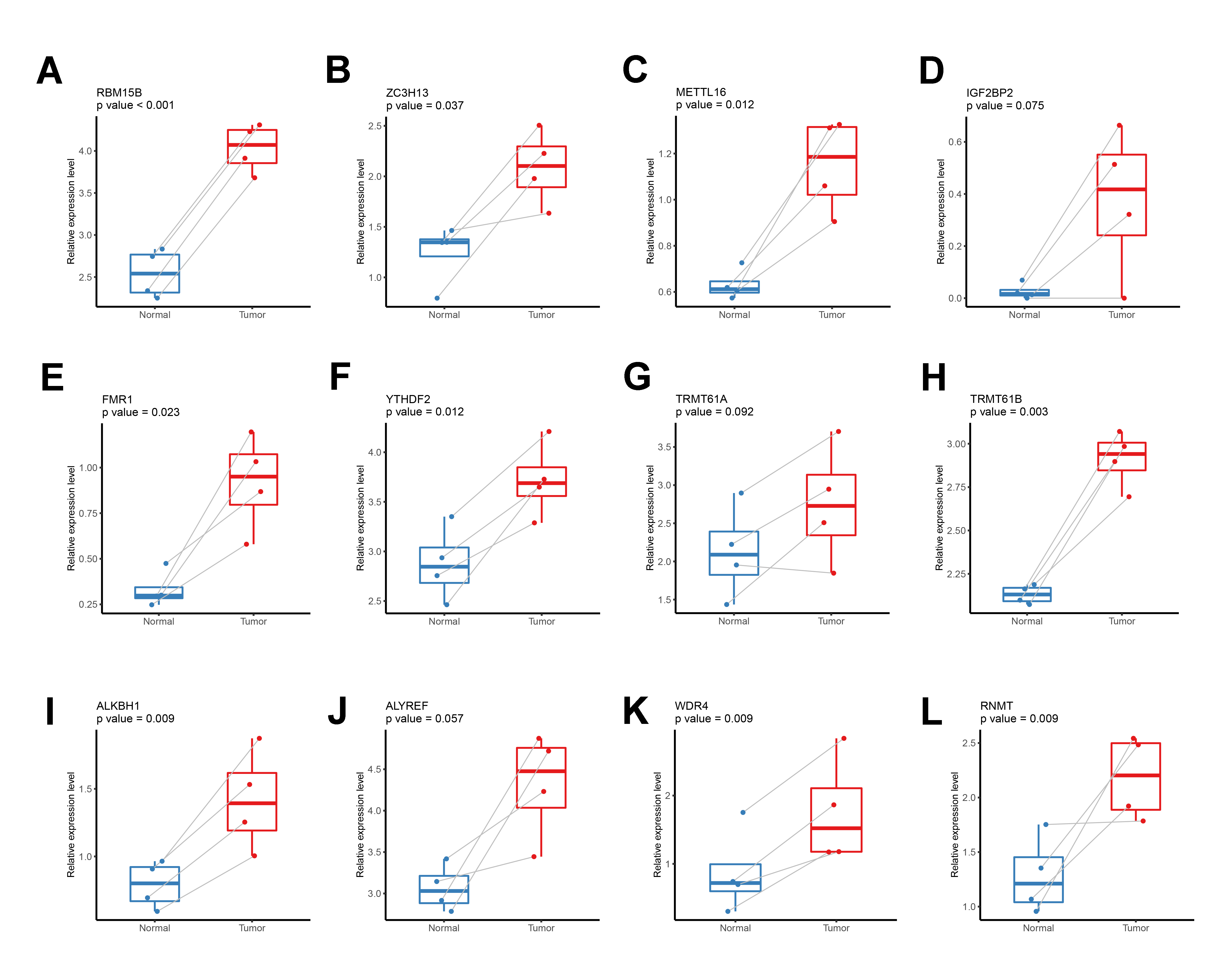

Supplement: Supplementary Figure 3 — The expression level of RNA modification regulators verified by sequencing data. (A–L) The expression level of RBM15B, ZC3H13, METTL16, IGF2BP2, FMR1, YTHDF2, TRMT61A, TRMT61B, ALKBH1, ALLYREF, WDR4 and RNMT based on the sequencing data. [file Image_3.jpeg]

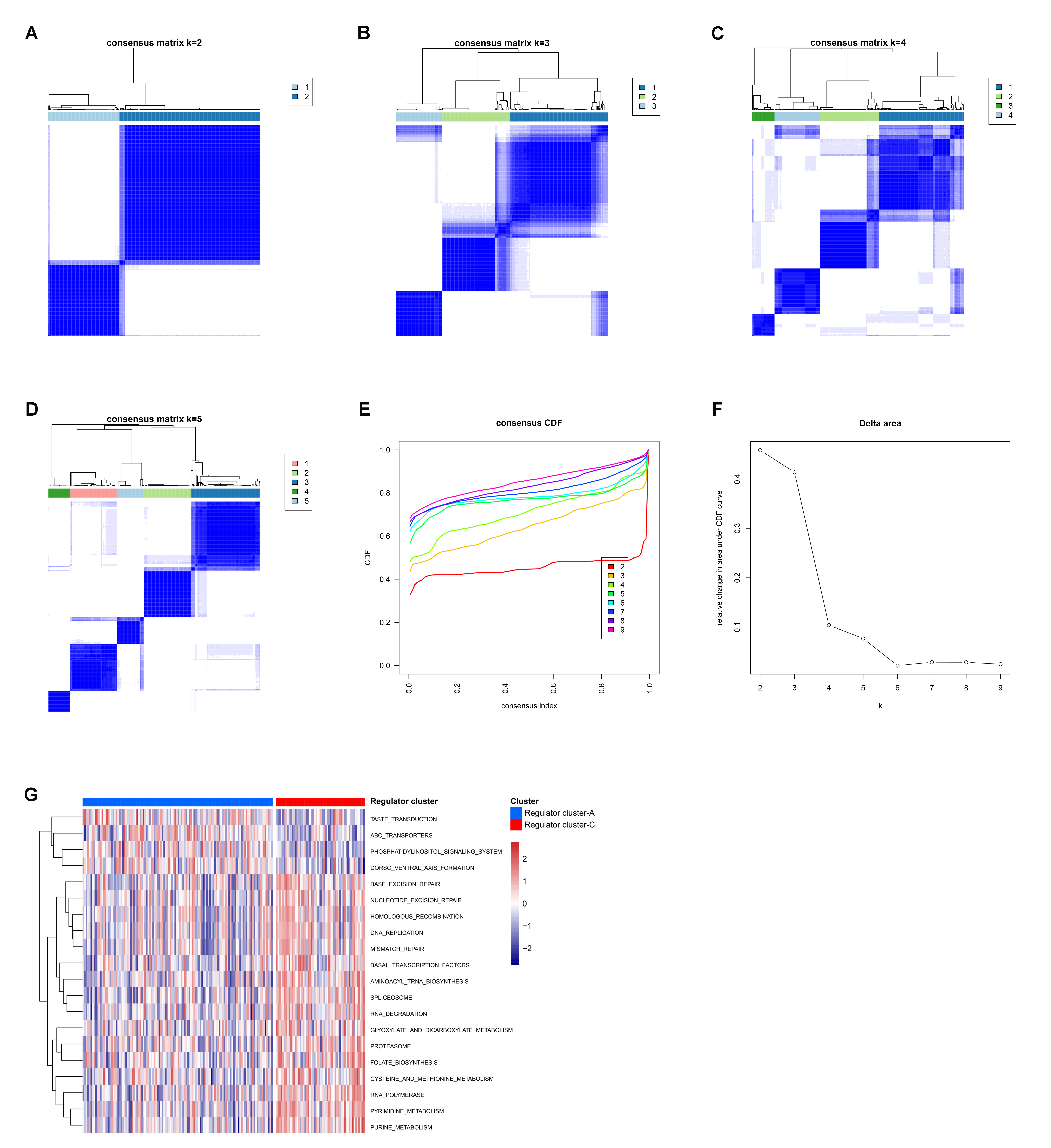

Supplement: Supplementary Figure 4 — Analysis of RNA modification Regulator Clusters in STS. (A–D) Consensus clustering based on RNA modification regulators (K = 2-5). (E) Consensus cumulative distribution function (CDF) Plot based on RNA modification regulators. (F) Delta area plot of consensus clustering based on RNA modification regulators. (G) The GSVA illustrating pathway enrichment among distinct Regulator Clusters. [file Image_4.jpeg]

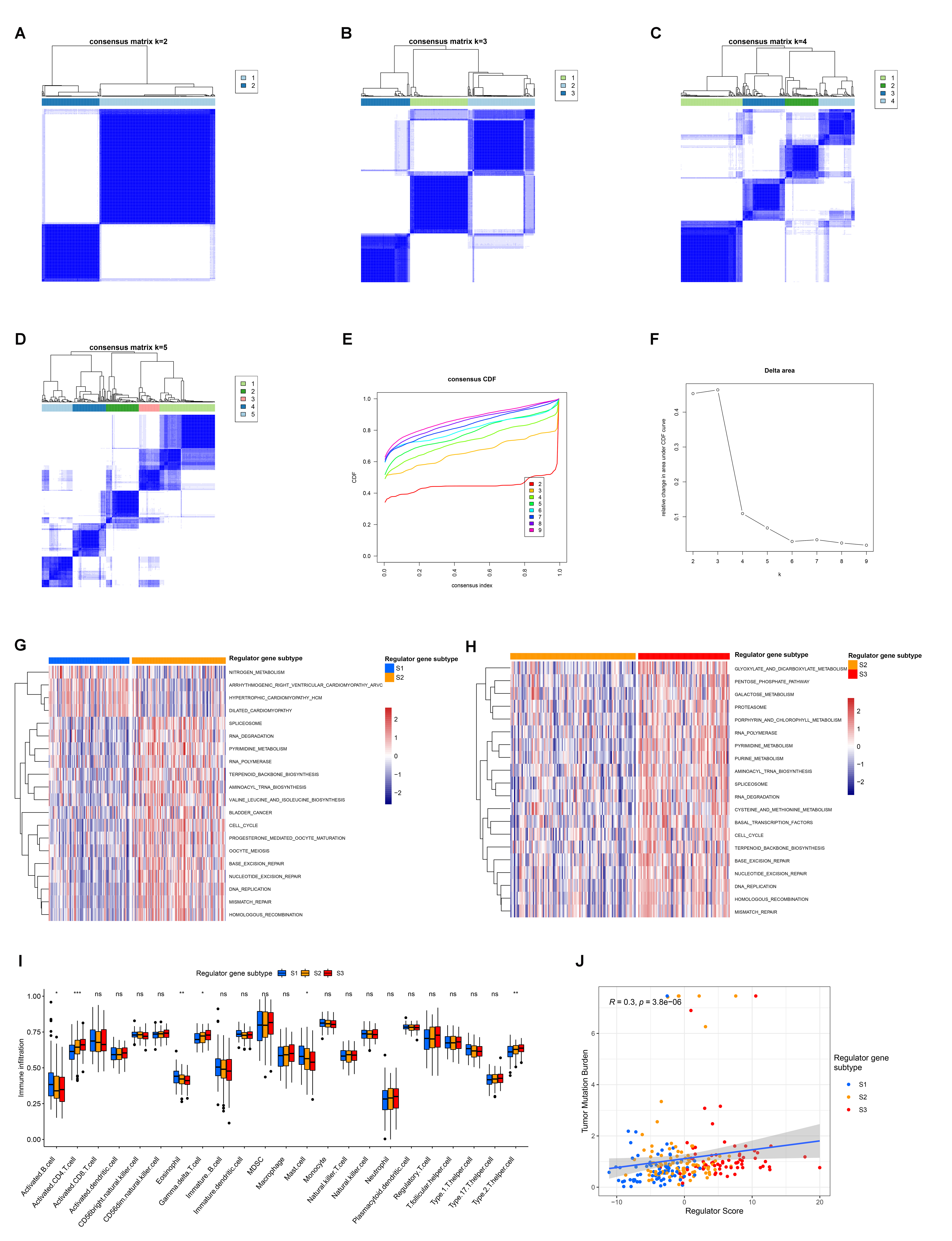

Supplement: Supplementary Figure 5 — Analysis of Regulator gene subtypes in STS. (A–D) Consensus clustering based on regulator-related DEGs (K = 2-5). (E) The CDF Plot based on the 54 RNA modification regulator-related DEGs. (F) Delta area plot of consensus clustering based on the DEGs. (G, H) The GSVA illustrating pathway enrichment among distinct Regulator gene subtypes. (I) The infiltration of immune cells within distinct Regulator gene subtypes. (J) Scatter plots depicting the positive correlation between TMB and Regulator Score in TCGA-SARC cohort. [file Image_5.jpeg]
